# The Impact of Obesity on the Resolution of Hypertension Following Adrenalectomy for Primary Hyperaldosteronism

**DOI:** 10.1007/s00268-023-07021-5

**Published:** 2023-07-14

**Authors:** Swathikan Chidambaram, Klaas Van Den Heede, Samir Damji, Karim Meeran, Jeannie Todd, Florian Wernig, Fausto Palazzo, Aimee N. Di Marco

**Affiliations:** 1 Department of Endocrine and Thyroid Surgery, Imperial College Healthcare NHS Trust, Hammersmith Hospital, Du Cane Road, London, W12 OHS UK; 2 Department of General and Endocrine Surgery, OLV Hospital Aalst, Aalst, Belgium; 3 Department of Medicine, Imperial College, London, UK; 4 Division of Surgery, Department of Surgery and Cancer, Imperial College, London, UK

## Abstract

**Background:**

This study aims to determine the impact of patient obesity on the resolution of hypertension and pill burden post-adrenalectomy for PA. Primary hyperaldosteronism (PA) is the most common cause of secondary hypertension that may be remedied with surgery (unilateral adrenalectomy). Obesity may independently cause hypertension through several mechanisms including activation of the renin–angiotensin–aldosterone pathway. The influence of obesity on the efficacy of adrenalectomy in PA has not been established.

**Methods:**

This is a retrospective analysis of prospectively collected data on patients undergoing adrenalectomy for PA at a single, tertiary-care surgical centre from January 2015 to December 2020. Electronic health records of patients were screened to collect relevant data. The primary outcomes of the study include post-operative blood pressure, the reduction in the number of anti-hypertensive medications and potassium supplementation burden post-adrenalectomy.

**Results:**

Fifty-three patients were included in the final analysis. There was a significant reduction in the blood pressure and the number of anti-hypertensive medications in all patients after adrenalectomy (*p* < 0.001). Of the 34 patients (64.2%) with pre-operative hypokalaemia, all became normokalaemic and were able to stop supplementation. However obese patients required more anti-hypertensive medications to achieve an acceptable blood pressure than overweight or normal BMI patients (*p* < 0.01). Multivariate logistic regression analysis showed that male gender and BMI were independent predictors of resolution of hypertension (*p* <0.01).

**Conclusion:**

Unilateral adrenalectomy improves the management of hypertension and hypokalaemia when present in patients with PA. However, obesity has an independent deleterious impact on improvement in blood pressure post-adrenalectomy for PA.

## Introduction

Primary hyperaldosteronism (PA) is characterised by the inappropriate secretion of aldosterone in the context of suppressed plasma renin levels [1]. The elevation in aldosterone leads to renal sodium retention and potassium excretion, resulting typically in hypertension and hypokalaemia. PA is the most common cause of secondary hypertension and may account for hypertension in 5–13% of patients [2]. In addition to the end-organ damage caused by hypertension, PA is associated with cardiovascular, renal, and metabolic complications [3]. In patients with lateralising PA (LPA), unilateral adrenalectomy is recognised as the standard treatment. However, the beneficial effects on hypertension following LPA are not entirely predictable due to additional variables with a long history of hypertension known to be associated with inferior results. However, other variables may also influence the efficacy of unilateral adrenalectomy on hypertension in the short and long term [4–6].

Previous studies have identified several variables such as age, body weight, presence of hypokalaemia, duration of hypertension, and number of anti-hypertensive drugs as predictive of resolution of hypertension post-adrenalectomy [5, 6]. Studies have proposed a correlation between obesity and hyperaldosteronism, including dysregulation of glucose homeostasis and metabolism as well as impaired insulin secretion due to hypokalaemia [7]. It is suggested that obese patients with a body mass index (BMI) above 30 are more likely to have higher levels of angiotensinogen, renin, aldosterone, and angiotensin-converting enzyme. Conversely, weight loss can decrease aldosterone levels. However, there is a paucity of data on the impact of obesity on the efficacy of adrenalectomy in the correction of hypertension. The results of studies have been mixed with some studies associating lower BMI with better surgical outcomes, while others dispute the relationship, pointing out that BMI does not correlate with plasma aldosterone levels [8, 9]. This study aims to understand the role of obesity in predicting the resolution of hypertension in patients undergoing unilateral adrenalectomy for LPA.

## Methods

### Study design and population

This is a retrospective analysis of prospectively collected data on fifty-three consecutive patients undergoing adrenalectomy for lateralising primary hyperaldosteronism at a tertiary referral centre. Patient demographics and pre-operative disease characteristics were extracted, including age, gender, BMI, pre-operative systolic blood pressure (SBP), pre-operative diastolic blood pressure (DBP), number of anti-hypertensive agents, use of potassium supplements and pre-operative biochemistry such as hypokalaemia, aldosterone-renin ratio (ARR), and aldosterone-cortisol ratio (ACR). BMI was categorised as normal (BMI < 25 kg/m^2^), overweight (BMI 25–30 kg/m^2^), obese (BMI 30–35 kg/m^2^), severely obese (BMI 35–40 kg/m^2^), and morbidly obese (BMI > 40 kg/m^2^). Details including operating time, approach and complications were extracted. Tumour characteristics such as maximum diameter on pre-operative imaging, laterality, and histological diagnosis were noted. Appropriate local ethical approval was obtained. The study has been reported using the STROBE Statement.

### Diagnosis of primary hyperaldosteronism

Patients with a potential diagnosis of PA were investigated with an adrenal panel of blood tests including renin and aldosterone levels. Those with a raised ARR in the absence of interfering medications and in the context of hypertension and/or hypokalaemia underwent a saline infusion test for biochemical confirmation. Cross-sectional imaging and adrenal venous sampling (AVS) are reserved for patients with biochemical confirmation and consented for an adrenalectomy in the event of lateralisation. All patients are discussed in the regional multidisciplinary team meeting at our institution after AVS and surgery for review of the histology.

### Outcomes and definitions

The primary outcomes of the study include the reduction in the number of anti-hypertensive medications, potassium supplementation, and BP measurements. The same definition of cure and improvement was used as in previously published work with cure defined as a non-invasive brachial BP of below 140/90 without any medications. If these criteria were not met, patients were considered ‘not cured’. Post-operative blood tests including ARR, sodium and potassium levels were also checked at follow-up in outpatient clinic. BP readings were non-invasive brachial measurements obtained at four time points, including time of diagnosis (before anti-hypertensive medications were prescribed); SBP on morning of the operation; 1 day post-operatively; and at follow-up typically three months after the operation. The number of anti-hypertensive medications and potassium supplements was obtained at the time of referral and at follow-up consultations. The maximum number of medications taken was used as the data point in cases where patients required up-titration of medications to control their BP. Secondary outcomes included post-operative complications, readmission to hospital, re-operation, and duration of stay in the hospital and intensive care unit.

### Statistical analysis

Statistical analysis was performed using Stata version 15.0 (Statacorp®, USA). All data were assessed for normality using the Shapiro–Wilks test and are expressed as means and standard deviations if they followed a parametric distribution. For evaluation of the impact of BMI on post-operative outcomes, a one-way Analysis of Variance was performed to detect statistical significance in the primary and secondary outcomes as well as baseline data between patients of five BMI categories. Multivariate logistic regression analysis was performed to detect statistically significant predictive factors between the “cured” and “not cured” cohorts, and stepwise regression to examine combinations of the parameters with p-values below 0.05. Cases with complete data were used for the final model to calculate the predicted probability of the outcome; draw the receiver operator characteristic curve; and calculate the area under the curve (AUC). Statistical significance was set at *p*  < 0.01 for all analyses.

## Results

### Demographic and pre-operative characteristics of patients

**Fig. 1:**
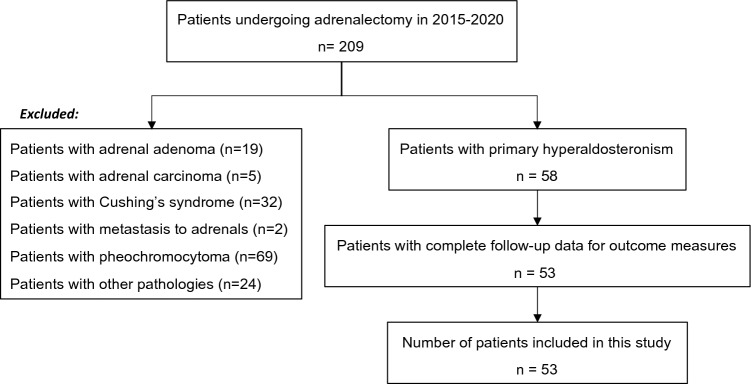
Analysis of registry for Inclusion of patients to study

From January 2015 to December 2020, 58 adrenalectomies were performed for PA (Fig. 1). One patient who also underwent surgery for a synchronous phaeochromocytoma was excluded from the analysis. At the time of analysis, 53 out of 57 patients had complete follow-up data on post-operative primary outcomes, specifically the BP and number of anti-hypertensive medications. Demographic and pre-operative characteristics of patients are shown in Table 1.

**Table 1:** Demographic and pre-operative characteristics of patients. All numerical data are given as mean and standard deviations, unless specified otherwise

Characteristic	Cured	Not cured	Significance
Sample size (%)	20 (37.7)	33 (62.3)	*p* > 0.01
Age at surgery in years	43.3 (11.4)	51.6 (9.2)	*p* > 0.01
Gender (%)			
Male Female	4 (7.5) 16 (30.2)	21 (39.6) 12 (22.7)	*p* = 0.0016
BMI in kg/m^2^	28.9 (7.3)	32.9 (4.9)	*p* = 0.025
Pre-operative systolic blood pressure in mmHg	135.0 (20.8)	146.0 (19.6)	*p* > 0.01
Pre-operative diastolic blood pressure in mmHg	82.9 (13.1)	90.8 (13.5)	*p* > 0.01
Pre-operative potassium supplementation (%)	10 (18.9)	24 (45.2)	*p* > 0.01
Pre-operative number of anti-hypertensive medications	2.4 (1.0)	2.9 (1.0)	*p* > 0.01
Aldosterone-cortisol ratio	3567 (2336)	3988 (2427)	*p* > 0.01
Aldosterone-renin ratio	21.2 (19.2)	36.3 (60.7)	*p* > 0.01
Adrenal vein sampling (% positive)	20 (37.7)	31 (58.5)	*p* > 0.01

### Operative and pathologic characteristics of patients

Fifty-one patients underwent a transperitoneal laparoscopic or retroperitoneoscopic unilateral adrenalectomy. Two patients underwent a transperitoneal procedure that was subsequently converted to an open procedure due to technical difficulty. There was no statistically significant correlation between BMI and the operative time (*r*^2^ = 0.0024), or involvement of the more senior surgeons (*p* = 0.49). There were no perioperative deaths or hospital readmissions for a surgical complication. Subgroup analysis showed no significant differences in operating times between surgeons or based on the operative approach. Operative and pathologic characteristics of patients are shown in Table 2.

**Table 2:** Operative and pathologic characteristics of patients. All numerical data are given as mean and standard deviations, unless specified otherwise

Characteristic	Cured	Not cured	Significance
Operative access (%) Laparoscopic Open Converted	20 (37.7) 0 0	31 (58.5) 0 2 (3.8)	*p* > 0.01
Operative approach (%) Retroperitoneal Transperitoneal	17 (32.1) 3 (5.7)	17 (32.1) 16 (30.2)	*p* > 0.01
Operative time in minutes	69.9 (24.7)	89.2 (34.9)	*p* > 0.01
Grade of principal surgeon as consultant (%)	17 (85.0)	25 (76.8)	*p* > 0.01
Final pathologic diagnosis (%) Hyperplasia Adenoma Angiomyolipoma	2 (3.8) 18 (33.9) 0	12 (22.6) 20 (37.7) 1 (1.9)	*p* > 0.01

### Outcomes after adrenalectomy

Complete follow-up data were available for 53 patients included in the final analysis (Supplementary file Table 1). There was a decrease in the post-operative SBP and DBP to 124 (SD = 12.1) and 87.7 (SD = 4.13) after the procedure (*p* < 0.001). Post-operative mean SBP was similar at 120 (SD = 9.9) and 128 (SD = 12.3) mmHg for the “cured” and “not cured” cohorts (*p* > 0.01). Post-operative DBP was higher at 105 (SD = 2.8) for the “not cured” group compared to the “cured” group at 76.7 (SD = 8.8), although this was not significant (*p* > 0.01). Of the thirty-four patients (64.2%) with pre-operative hypokalaemia, there were no patients requiring potassium supplementation after the operation for both “cured” and “not cured” groups. Accordingly, there was also normalisation of the ARR values for all patients, reflecting a biochemical resolution of hyperaldosteronism. There was a 51% reduction in the number of anti-hypertensive medications from 2.74 (SD = 0.14) to 1.36 (SD = 0.19) post-adrenalectomy. Post-operatively, the average number of medications used by the 33 patients who were not cured was 2.18 (SD = 1.13) (*p* < 0.01). There was no post-operative mortality and morbidity resulting in hospital readmission.

### Impact of BMI on post-operative outcomes

**Fig. 2:**
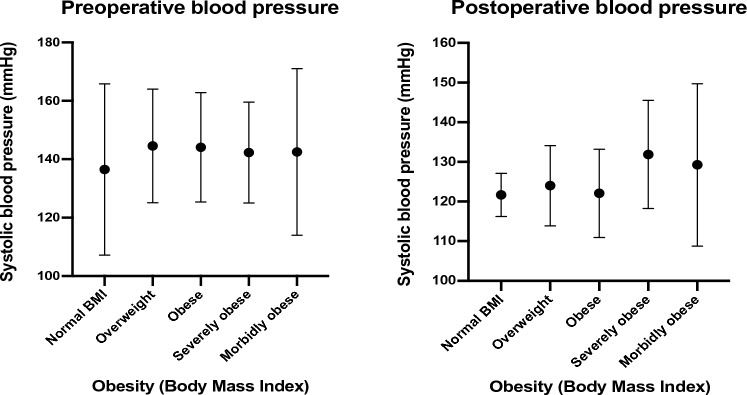
Effect of BMI on pre- and post-operative blood pressure

**Fig. 3:**
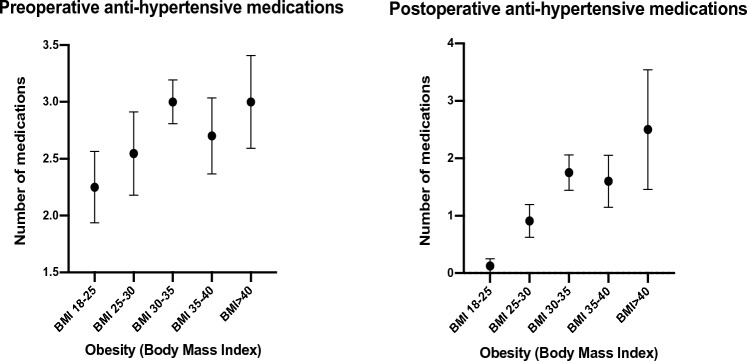
Effect of BMI on number of anti-hypertensive medications

Fifty-three patients were included in the final analysis related to BMI (Supplementary file Table 2). Two patients who experienced significant weight loss after bariatric surgery were excluded. The mean BMI of the cohort was 31.7 kg/m^2^ (SD = 6.3), with a high predominance of overweight or obese patients. Stratifying patients based on their BMI, there was no statistically significant difference in the relevant baseline pre-operative, operative, or pathologic characteristics (*p* > 0.01). There was no correlation between BMI and post-operative mortality, morbidity-related hospital admissions, or post-operative blood pressures (*p* > 0.01) (Fig. 2). However, patients with a BMI > 30 kg/m^2^ remained on a greater number of anti-hypertensive medications than lean or overweight patients (*p* < 0.01) (Fig. 3).

### Predictors of cure

**Fig. 4:**
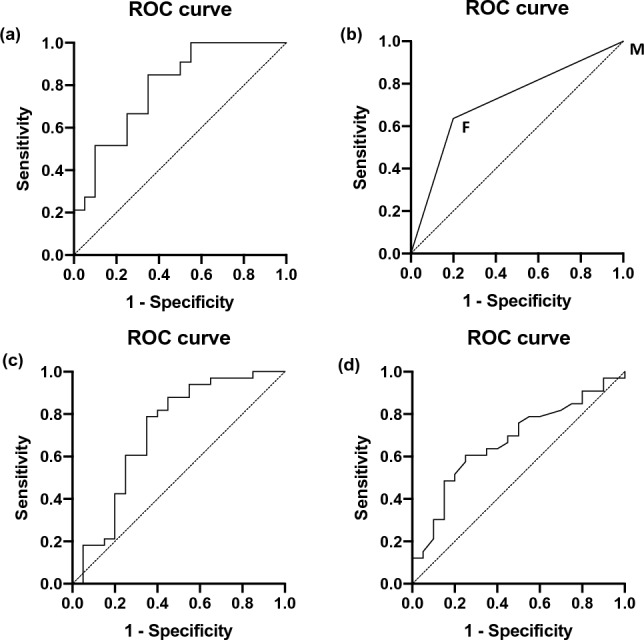
Multi-logistic regression analysis of predictive factors of the cured patients; **a** ROC characteristic analysis of gender, BMI, and operating time; **b** ROC characteristic analysis of gender; **c** ROC characteristic analysis of BMI; **d** ROC characteristic analysis of operating time

**Fig. 5:**
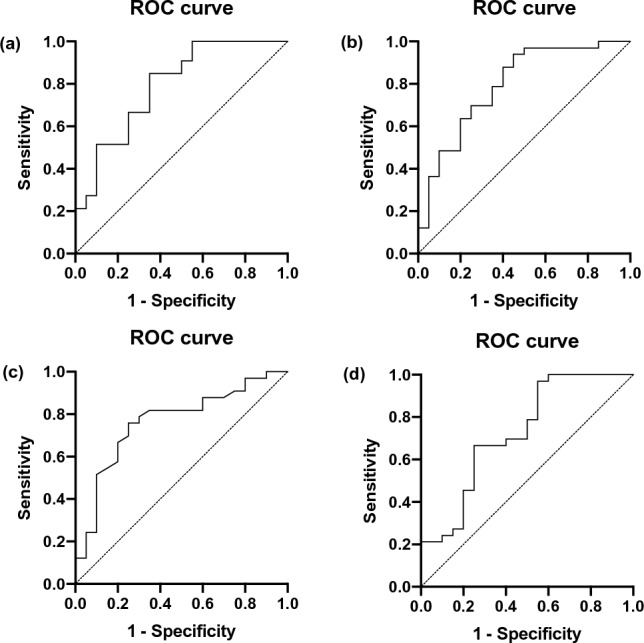
Multi-logistic regression analysis of predictive factors of the cured patients; **a** ROC characteristic analysis of gender, BMI, and operating time; **b** ROC characteristic analysis of gender and BMI; **c** ROC characteristic analysis of gender and operating time; **d** ROC characteristic analysis of BMI and operating time

Initial multivariate analysis showed three variables (BMI, gender, and operative time) to be statistically significant between patients who were cured and not cured (*p* < 0.01). Using stepwise regression analysis, the odds ratio for BMI, gender, and operating time was 4.56 (CI: 1.18 to 20.6), 1.207 (CI: 1.10 to 1.25) and 1.02 (CI: 0.99 to 1.05) (*p* < 0.01), respectively. For individual factors, the AUC was 0.72 for gender (CI: 0.58 to 0.86, *p* = 0.0082)); 0.72 for BMI (CI: 0.56 to 0.87, *p* = 0.0087); and 0.67 for operating time (CI: 0.52 to 0.82, *p* = 0.0373), indicating that patients of male gender or a higher BMI are less likely to be cured of hypertension post-operatively (Fig. 4). Notably, the AUC was 0.79 (CI: 0.66 to 0.92, *p* = 0.0004) for all three factors; 0.79 (CI: 0.67 to 0.92, *p* = 0.0004) for gender and BMI only; 0.76 (CI: 0.63 to 0.89, *p* = 0.0014) for gender and operating time only; and 0.73 (CI: 0.58 to 0.87, *p* = 0.0059) for BMI and operating time only (Fig. 5).

## Discussion

The optimal management of lateralising primary hyperaldosteronism is adrenalectomy to remove the source of aldosterone hypersecretion [10, 11]. However, there is wide variability in the biochemical and clinical outcomes, with previous studies reporting a cure rate of pill-free hypertension below 50% and partial response in most patients [12]. The PASO study is the largest international collaborative effort to identify factors that influence clinical outcomes after adrenalectomy, including obesity [4]. Since then, other smaller retrospective studies have reported contradictory outcomes. These studies are limited by significant heterogeneity given the long timescale of data collection during which the nuances of clinical practice changes, and the low case volume per centre. Thus, to our knowledge, this study, undertaken in the UK’s highest volume centre, is the first that addresses the impact of obesity on the resolution of hypertension after adrenalectomy. As expected, hypokalaemia resolved in all patients. Furthermore, there was improvement either in the blood pressure or number of post-operative anti-hypertensive medications in all patients. BMI was an independent predictor of clinical reduction in hypertensive pill burden although it does not significantly affect post-operative BP, that is to say that although all patients had lower blood pressures compared to initial presentation, patients with a higher BMI achieved this by remaining on several medications, and hence a similar pill burden.

Obesity is a systemic disease linked to metabolic manifestations such as type 2 diabetes mellitus, hypertension and dyslipidaemia [13]. Adipocytes secrete molecules such as adiponectin, CTRP1, leptin, osteopontin, and resistin, which may stimulate aldosterone production in addition to the aldosterone-secreting adenoma [14]. Insulin and leptin result in sympathetic stimulation and reinforce an already hyperactive renin-angiotensin axis [15]. In turn, aldosterone activates mineralocorticoid receptors and causes maturation of adipocytes, resulting in a positive feedback cycle. In the long term, persistently high aldosterone levels cause vascular remodelling and high BP similar to essential hypertension [16]. After the adrenal gland is removed, although there is no aldosterone from the tumour, the persistence of adipocyte-related aldosterone secretion independent of renin may explain the unresolving hypertension [17]. While a direct correlation between BMI and plasma aldosterone levels has been shown, there is little evidence for a similar association after tumour resection. Hence, further biochemical studies in patients with regular long-term follow-up are necessary to confirm this.

Several studies have identified age, sex, ARR, ACR, duration of hypertension, nodule size, number of anti-hypertensive medications, and operating time as significant contributors to outcomes of adrenalectomy [4, 5]. In our cohort, we identified BMI and gender as the strongest predictors. Of these, BMI is the only modifiable factor that can be optimised pre-operatively. This adds to the importance of weight loss in managing these patients. Contrary to previous work, we did not detect significant input from the other factors although this may be due to smaller sample sizes [18–20]. Notably, we did not have full data on the duration of hypertension which is likely a significant predictive factor. However, as a real-world study in which patients are referred at a variety of time points in the course of their PA, this study methodology and results may assist with surgical decision-making. Previous studies have highlighted that bariatric surgery induced weight loss induces a progressive decrease in PAC independently of PRA and sodium excretion. There is still minimal evidence to inform us on the timing of bariatric interventions, specifically if combined adrenalectomy and bariatric procedures are advantageous to staged procedures; or if bariatric surgery can lead to cure without a subsequent need for adrenalectomy. These important considerations remain an area for further research.

In our institution the decision regarding the relative timing of adrenal and bariatric surgery is made between the adrenal and bariatric multidisciplinary teams. The main driver for the order of procedures is the severity of the hypertension versus the technical challenge of adrenalectomy in the obese patient. It is for this reason that three patients in the study group underwent adrenalectomy first, to assist with control of the hypertension, followed by bariatric surgery second. The option of performing combined bariatric and adrenal surgery has thus far, not been explored in our centre due to a combination of logistical factors and concern regarding the potential for more complex post-operative management.

A consistent feature in most patients was depleted potassium levels alongside high aldosterone levels. 64.2% of the cohort had pre-operative hypokalaemia that required supplementation and potassium-sparing anti-hypertensive medications. Pre-operative potassium levels did not correlate with resolution of hypertension. Notably, all patients had resolution of hypokalaemia and were not on any potassium supplementation at follow-up. Although plasma aldosterone and renin levels were not collected for all patients, there was a reduction in these hormone levels where data were available regardless of age, gender, or BMI. Hence, adrenalectomy predictably leads to a biochemical resolution although the clinical reflection of this can be masked by other factors [21]. Again, this adds evidence to the postulate that persistent hypertension is due to mediators outside of the renin-angiotensin axis.

A majority (64.2%) of the patients were classified as obese (Supplementary file Table 2). Three patients underwent bariatric surgery post-adrenalectomy. These patients used more anti-hypertensive medications; were hypertensive despite multiple medications; and remained hypertensive in the interim between adrenalectomy and bariatric surgery. Following bariatric surgery, patients experienced significant weight loss, followed by marked improvements in BP and reduction in medications. Previous studies reported the association between weight loss and reduction in BP in obese patients undergoing surgery or medical therapy [22]. However, most studies excluded patients with secondary hypertension, resulting in an absence of sufficient evidence to outline the utility of weight loss in patients with primary hyperaldosteronism [23]. Based on these cases, it may be useful to identify obese patients who may qualify for bariatric surgery and time it so that the potential for resolving hypertension is increased.

Our study has several strengths. It is a consecutive series performed at arguably the highest volume tertiary centre in the UK and is the first to explore the impact of obesity on the resolution of hypertension after adrenalectomy. We also matched for confounding factors known to affect the primary outcome, including age, gender, operative time, and grade of the principal surgeon. Nevertheless, there a few limitations to our work. Firstly, this is a single-centre retrospective study with less generalizability. The small sample size may introduce type II errors. Follow-up for most patients was limited to three months, hence long-term effects of surgery could not be determined but will be reported in future work [24]. Furthermore, we could have incorporated a more holistic assessment of visceral obesity based on anthropometric measurements and pre-operative cross-sectional imaging. Although we matched patients for demographic and operative variables, we did not account for the confounding effects of other metabolic parameters including cardiovascular function and type 2 diabetes mellitus. Thus, future work should be aimed at large-scale, multi-centre, prospective cohort studies with longer follow-up to fully investigate the impact of obesity and closely related factors on hypertension post-adrenalectomy.

## Conclusion

This study shows that BMI significantly influences the post-operative anti-hypertensive pill burden in patients undergoing adrenalectomy. While surgery certainly reduces the blood pressure, obese patients require medications to sustain this effect. Obesity is a modifiable risk factor that could be optimised pre-operatively to obtain better outcomes and may inform patient selection. Regardless of other factors, surgery consistently resolves hypokalaemia, reflecting a biochemical resolution of hyperaldosteronism. Further work is required to confirm this on a larger scale. This data will be useful when discussing the predicted personalised outcomes during the consenting process so patients are counselled appropriately on the prospects of improvement.

## Author contribution

ADM conceived the idea for the study. ADM and SC designed the study. SC, SD, and KVDH were involved in data collection. SC and KVDH analysed and interpreted the data. ADM, FFP, KVDH, SD, and SC drafted the manuscript. SC is study guarantor. All authors reviewed the final manuscript, agreed to be accountable for all aspects of the work, and approved the final manuscript for submission. The corresponding author attests that all listed authors meet authorship criteria and that no others meeting the criteria have been omitted.

## Declarations

### Patient and public involvement

This research was done without patient involvement. Patients were not invited to comment on the study design and were not consulted to develop patient relevant outcomes or interpret the results. Patients were not invited to contribute to the writing or editing of this document for readability or accuracy. Patient involvement with and understanding of the outcomes is important and it is planned that the outcomes will be disseminated via patient groups for PA and for bariatric surgery.
